# Intravascular lithotripsy followed by angioplasty and stenting for severely calcified carotid artery stenosis: technical note and case series

**DOI:** 10.3389/fneur.2025.1634201

**Published:** 2025-09-03

**Authors:** Macy Mitchell, Leonard H. Verhey, Naveen Taylor, Andrea Sewell, Andres Restrepo Orozco, Paul Mazaris

**Affiliations:** ^1^Department of Medicine, College of Human Medicine, Michigan State University, Grand Rapids, MI, United States; ^2^Department of Neurosurgery, Corewell Health West, Grand Rapids, MI, United States

**Keywords:** balloon angioplasty, high-energy shock wave, carotid stenosis, lithotripsy, case series

## Abstract

**Purpose:**

In patients with circumferential heavily calcified carotid stenosis, conventional carotid artery stenting (CAS) has its limitations, and carotid endarterectomy may be contraindicated. Intravascular lithotripsy (IVL) as an adjunct to CAS may be well suited for this subset of patients. We provide a technical report and series of five patients with severe, calcified carotid stenosis who underwent adjunctive IVL with CAS at our tertiary center.

**Methods:**

All patients who underwent CAS with adjunctive IVL for severe carotid calcific stenosis at our center were included. All data were extracted from the electronic medical record and the departmental database. Data were synthesized in accordance with Preferred Reporting of Case Series in Surgery (PROCESS) guidelines.

**Results:**

Five patients underwent CAS with adjunctive IVL for severe carotid stenosis between November 2022 and August 2024. The mean age at time of the procedure was 78.8 (SD 4.9); 80% were male. Patients presented with symptomatic stenosis, ranging from 50 to >95%. Symptoms included visual deficits (60%), hemiparesis (40%), facial droop (40%), and dysarthria (40%). All patients underwent post-procedural duplex ultrasound, which demonstrated resolution or significant reduction of stenosis. There were no clinical thromboembolic events. All patients have been followed in our comprehensive stroke program. Two patients died from causes unrelated to the procedure.

**Conclusion:**

Adjunctive intravascular lithotripsy in patients with severe, circumferential calcified stenosis is a novel technique that has shown promising preliminary results. This application of intravascular lithotripsy warrants further investigation.

## Introduction

Carotid angioplasty and stenting (CAS) remains challenging in patients with heavily calcified carotid stenosis, particularly those with circumferential calcification. Specifically, the radial force of current carotid stent technology is often insufficient to overcome the calcification, making it recalcitrant even to high-pressure balloon angioplasty (1, 2). Angioplasty typically addresses only the superficial, luminal-facing calcified plaque, and both angioplasty and stenting in this subset of patients are associated with thromboembolic events, carotid arterial dissection, and even carotid artery rupture (3). While carotid endarterectomy (CEA) remains a viable treatment strategy for some patients with severe, circumferentially calcified carotid stenosis, not all patients are suitable for general anesthesia or awake CEA, necessitating alternative therapeutic approaches.

Medical management, including lipid-lowering, antihypertensive, and antiplatelet agents, blood sugar control, and lifestyle modifications, is an appropriate strategy for treating patients with carotid artery stenosis. In symptomatic patients with ≥70% stenosis, revascularization is associated with a reduced risk of ischemic stroke. Additionally, select patients with symptomatic mild to moderate stenosis (50–69%) may also benefit from revascularization (4).

Intravascular lithotripsy (IVL) is an endovascular adjunct for patients with severe, heavily calcified carotid stenosis who are not candidates for carotid endarterectomy (2). This technique has been used in coronary and peripheral artery patient populations (5). Shockwave lithotripsy (Shockwave Medical Inc., Johnson & Johnson MedTech, Santa Clara, CA, United States) emits sonic pressure waves within the arterial lumen that fracture heavily calcified plaques, rendering them more amenable to angioplasty and stenting. This therapy has enabled more favorable navigation across stenoses without thromboembolic events, enhanced luminal expansion, improved stent-vessel wall apposition, and improved clinical outcomes in the cardiac and peripheral artery disease literature (5). The application of this technology to carotid artery stenosis remains largely exploratory and off-label. A small number of case reports illustrating the use of IVL in severe, calcified carotid stenosis have recently been published in the neurointerventional literature.

We provide a technical report and clinical series of five patients with symptomatic severe, calcified carotid stenosis who underwent adjunctive intravascular lithotripsy with carotid artery stenting at our tertiary center.

## Methods

Informed consent for this case series was waived. Procedural consent for digital subtraction angiography with carotid angioplasty and stenting, including the intended off-label use of IVL, was obtained from all patients. The study adhered to the Preferred Reporting of Case Series in Surgery (PROCESS) guidelines (6). Data presented in this series are available from the corresponding author upon reasonable request.

### Inclusion criteria and data collection

All patients who underwent angioplasty with IVL and carotid stenting for severe carotid calcific stenosis at our center between November 2022 and August 2024 were included. The degree of carotid stenosis was quantified on CTA according to the North American Symptomatic Carotid Endarterectomy Trial (NASCET) criteria (7). All patients had symptomatic disease with >50% stenosis. Patients were also deemed to be at an unacceptably high risk for general anesthesia based on the comorbidity profile. Data were extracted from electronic medical records and our departmental neurointerventional database. Intraprocedural and postprocedural complications were recorded. Available clinical data recorded at any follow-up time point following the procedure were captured.

### Endovascular procedure

Patients were placed on dual antiplatelet therapy (DAPT) prior to intervention and were continued on DAPT for several weeks following the procedure. At our institution, for patients undergoing carotid artery stenting, DAPT is routinely initiated without a pre-procedural P2Y12 assay. Given the high flow of the carotid artery, our decision to proceed to stent placement is not dependent on the patient’s response to clopidogrel. The procedure was conducted under conscious sedation. Under ultrasound guidance, a 7-French (F) sheath was inserted into the right distal radial artery to establish arterial access. A Benchmark BMX^®^ 81 (Penumbra, Alameda, CA, United States) catheter was then navigated into the common carotid artery using a 5F Simmons Select catheter and a 0.035-inch GLIDEWIRE^®^ (Terumo Interventional, Somerset, NJ, United States). At this point, anticoagulation was initiated for the remaining duration of the procedure. The Select catheter and GLIDEWIRE^®^ were removed. An Emboshield^®^ NAV6 distal embolic protection device and the Shockwave M5 + IVL balloon were coaxially navigated into the common carotid artery, and the embolic protection device was deployed within the distal cervical ICA. The IVL balloon was inflated to 4 atm merely to hold the balloon in place. The balloon measured 60 mm in length and 7 mm in diameter. Inside the balloon, a vapor bubble expands and contracts, creating sonic pressure waves. The device delivers 30 pulses per cycle. Typically, 1–2 cycles were performed at the discretion of the neurosurgeon, based on the severity of calcification and the degree of luminal dilation achieved. These sonic pulses do not dislodge plaque but merely create fissures within the calcified plaque, thereby restoring the conformability of the carotid artery. Once satisfied with the degree of dilation, stenting was performed using either an Xact^™^ or Precise^®^ carotid stent. Post-processing with balloon angioplasty was performed as needed. The distal embolic protection device was retrieved. Final angiographic runs were taken of the cervical common carotid and internal carotid arteries as well as the cerebral circulation to confirm patency. The radial artery access site was secured with a PreludeSYNC DISTAL^™^ band (Merit Medical, South Jordan, UT, United States). Patients were monitored as per routine with continuous blood pressure readings for at least 1 day in a stepdown unit prior to discharge. All patients underwent carotid duplex ultrasonography post-procedure prior to discharge.

## Results

### Case 1

**Figure 1:**
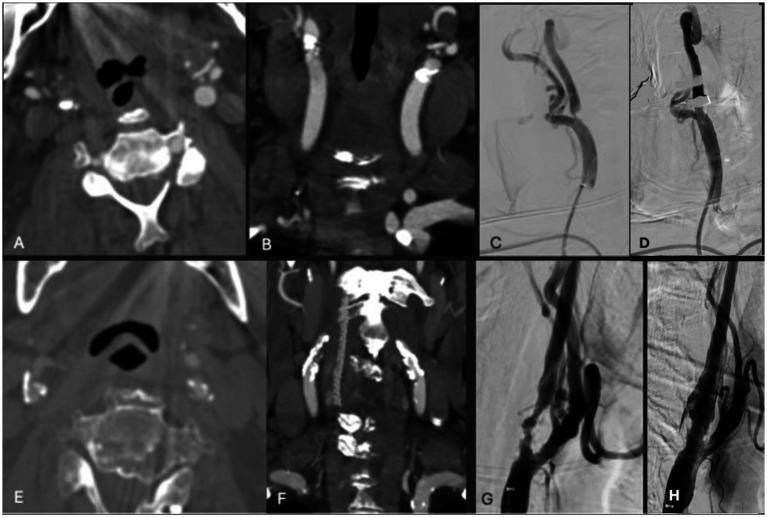
**(A,B)** Axial and coronal CTA demonstrating right ICA circumferential calcified stenosis. Catheter angiography demonstrating **(C)** severe R ICA stenosis with **(D)** significant improvement in caliber and flow within right ICA following lithotripsy, stent and angioplasty. **(E,F)** Axial and coronal CTA demonstrating right ICA circumferential calcified stenosis. Catheter angiography demonstrating **(G)** severe R ICA stenosis with **(H)** significant improvement in caliber and flow within right ICA following lithotripsy, stent and angioplasty.

A patient in their 80s presented with left homonymous hemianopia and dizziness (Table 1). CTA revealed greater than 80% stenosis of the right ICA. Associated right hemispheric watershed punctate infarcts were noted on diffusion-weighted MRI. The level of the carotid bifurcation with severely calcified stenosis was located high in the neck and deemed surgically accessible; therefore, CAS with concomitant IVL was recommended over CEA. One episode of sonic pulses was released over 1 min, with approximately one sonic pulse cycle per second (Figure 1). The patient was neurologically intact following the procedure. Post-angiogram ultrasound showed a peak systolic velocity (PSV) of 106 cm/s in the ICA/stent with no significant stenosis visualized within the stent. The PSV ICA/CCA ratio was 1.7 with a normal vertebral artery waveform. Following the stenting procedure, the patient experienced transient hypotension followed by left-sided drift. MRI was completed at that time, showing small foci of acute infarctions in the right frontal lobe and right corona radiata. It was found that the P2Y12 reactive unit assay suggested they were non-responders, and therefore, clopidogrel was stopped and ticagrelor was initiated. The patient was discharged to inpatient rehabilitation after an 8-day hospital stay. At the 3-week follow-up, they had no visual deficit, and the left-sided weakness had resolved. The patient was then transferred to an outside hospital for further care.

**Table 1:** Summary table compares baseline demographics, clinical presentation, and procedural outcomes, including age, sex, presenting symptoms, CT angiography findings, number of IVL pulses delivered, and post-procedural peak systolic velocities as measured using duplex ultrasound.

Patient	Age, sex	Presenting symptoms	CTA	Shockwave episodes	Peak systolic velocity (cm/s)
Case 1	80 y/o, M	Left homonymous hemianopia, dizziness	>80% stenosis RICA	1	RICA: 106 R ICA/CCA: 1.7
Case 2	75 y/o, M	Right visual disturbance	90% stenosis RICA	2	RICA: 102 R ICA/CCA: 1
Case 3	81 y/o, M	Progressive confusion, left visual field deficit	90% stenosis RICA	1	RICA: 100 R ICA/CCA: 1.2
Case 4	72 y/o, F	Left hemiplegia, left facial droop, dysarthria	95% stenosis RICA	2	RICA: 110 R ICA/CCA: 1.1
Case 5	86 y/o, M	Left facial droop, left-sided paresis, dysarthria	50–60% stenosis RICA	2	RICA: 100 R ICA/CCA: 1.4

### Case 2

A patient in their 70s presented with a transient ischemic attack (TIA) that manifested as a right eye vision disturbance. CTA revealed 90% occluded right ICA. MRI was obtained and showed no acute infarct. Given the patient’s concurrent advanced heart failure, history of coronary artery bypass graft, and cervical laminectomy and instrumented fusion, they were deemed unfit for general anesthesia and carotid endarterectomy. IVL with stenting was performed with 2 episodes of pulses (Figure 1). Post-angiogram ultrasound showed a PSV of 102 cm/s in the right ICA with a PSV ratio of 1 in the ICA/CCA. There were no post-procedural complications, and the patient was discharged in stable condition 1 day following the procedure. At the 3-week follow-up, the patient was clinically stable with no observed or reported neurological deficits, aside from a baseline mild facial droop. However, the patient unfortunately died 7 months following the procedure secondary to an acute exacerbation of chronic heart failure.

### Case 3

**Figure 2:**
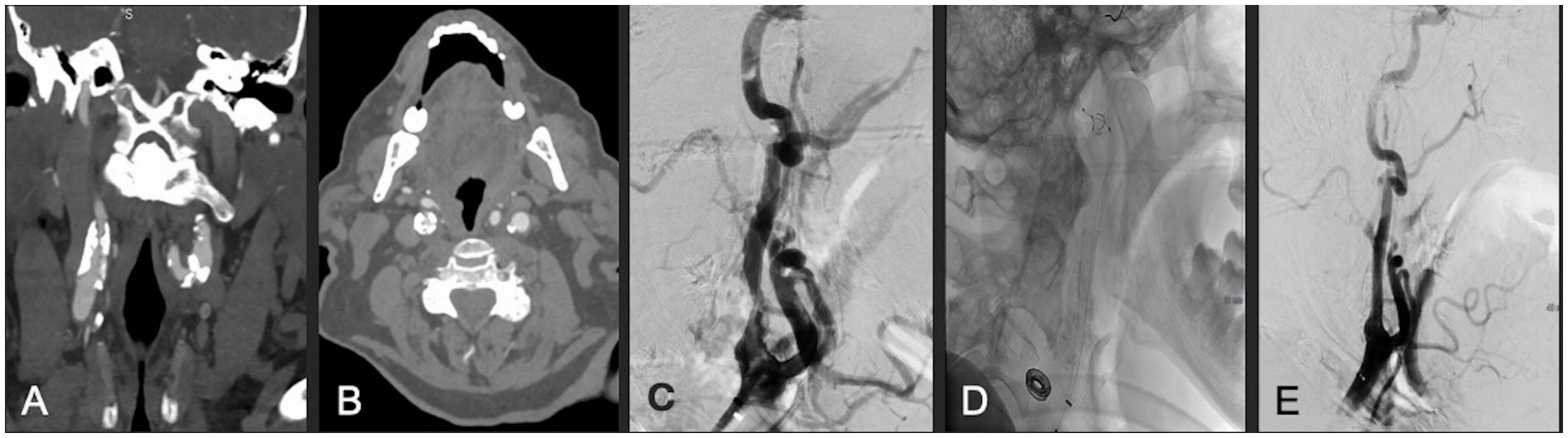
**(A,B)** Coronal and axial CTA demonstrating right ICA circumferential calcified stenosis. Catheter angiography demonstrating **(C)** severe ICA stenosis, **(D)** carotid stent and distal embolic protection device in place, and **(E)** significantly improved caliber and flow within right ICA following lithotripsy, stent and angioplasty.

A patient in their 80s presented with progressive confusion as well as blurriness in the left visual field. They were found to have 90% stenosis of the right ICA with a fetal origin posterior communicating artery, arising from anterior circulation, with an associated right occipital infarct. CTA demonstrated extensive calcification of the right carotid bulb, and a very distally located bifurcation that was deemed not amenable to surgery. Therefore, the patient was successfully treated with IVL and stenting via one episode of sonic pulses (Figure 2). Post-angiogram ultrasound findings showed PSV of 100 cm/s, a peak systolic ICA/CCA velocity ratio of 1.2, and less than 50% stenosis in the right ICA. The patient had a 7-day hospital stay without post-procedural complications, after which they were discharged to a rehabilitation hospital. At 1-month follow-up, the patient’s confusion had resolved; however, a left inferior homonymous quadrantanopia persisted. Unfortunately, the patient was diagnosed with lung cancer and died 19 months following the procedure secondary to acute hypoxic respiratory failure.

### Case 4

**Figure 3:**
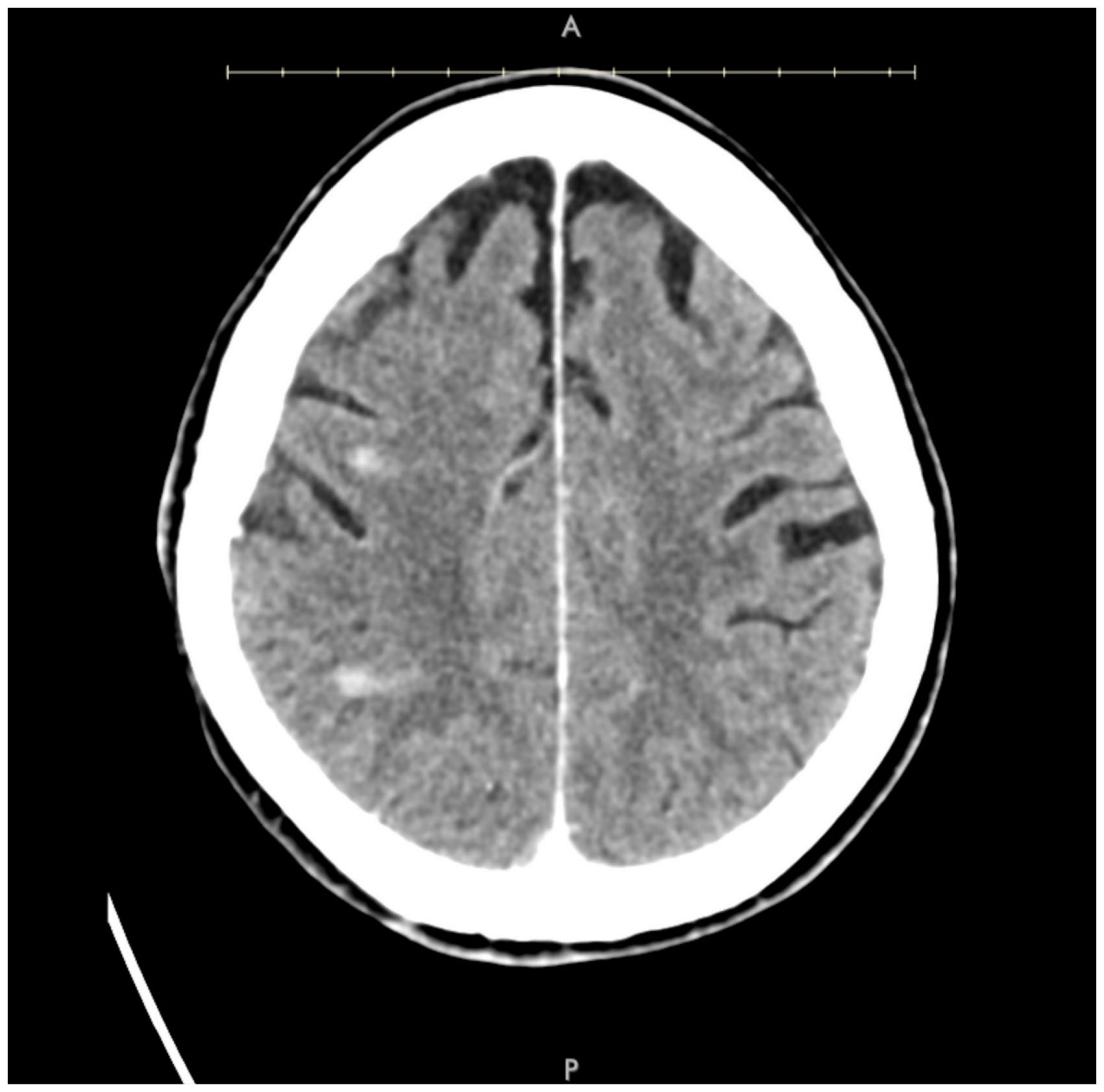
Axial post-contrast CT demonstrating small areas of enhancement within the right MCA territory.

**Figure 4:**
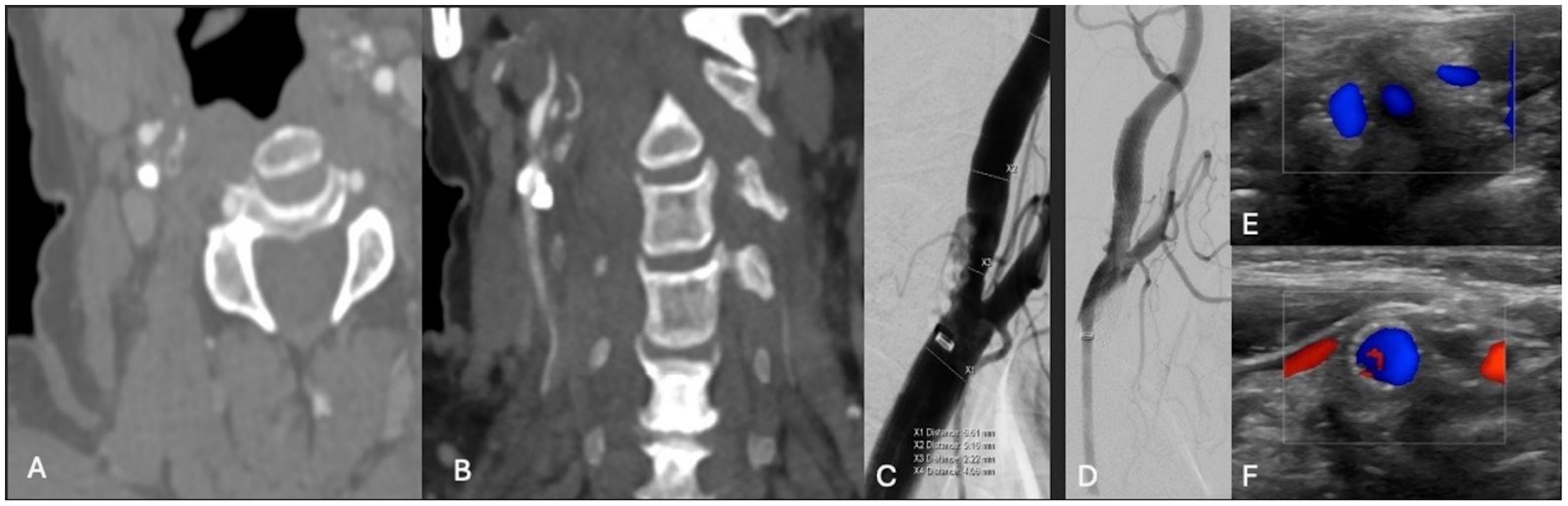
**(A,B)** Axial and coronal CTA demonstrating right ICA circumferential calcified stenosis. Catheter angiography demonstrating **(C)** severe R ICA stenosis. **(D)** Catheter angiography demonstrating improved caliber and flow following lithotripsy, stenting and angioplasty. **(E,F)** Duplex ultrasonography of the right carotid bulb and ICA following lithotripsy, stent and angioplasty demonstrating stent patency and normal peak systolic velocity.

A patient in their 70s presented with left-sided hemiplegia, left facial droop, and dysarthria. CTA demonstrated greater than 95% stenosis in the right ICA. Computed tomography imaging showed scattered right middle cerebral artery (MCA) infarcts (Figure 3). The patient was at high risk for surgery secondary to numerous medical comorbidities, including hypertension, peripheral vascular disease s/p peripheral bypass with subsequent thrombosis, left above-the-knee amputation, and bilateral renal stenosis. Thus, they underwent IVL and CAS with 2 episodes of sonic pulses (Figure 4). Right-sided radial access was attempted but unsuccessful in this patient; ultimately, femoral arterial access was established. Post-procedurally, the patient developed a right radial pseudoaneurysm and a hematoma at the right femoral access site, which was associated with acute blood loss anemia (hemoglobin at 6.6 g/dL) that normalized after transfusion. No surgical intervention for the femoral access site was required. Post-angiogram ultrasound findings showed PSV of 110 cm/s in the right CCA and 122 cm/s in the right ICA with a peak systolic ICA/CCA velocity ratio of 1.1 and no stenosis in the right ICA. The patient’s hospital stay was 9 days, followed by 2 weeks of inpatient acute rehabilitation. The patient did not establish neurosurgical follow-up; however, at the time of discharge from acute rehabilitation, the patient had mild weakness in the left upper extremity with improved finger dexterity and coordination.

### Case 5

**Figure 5:**
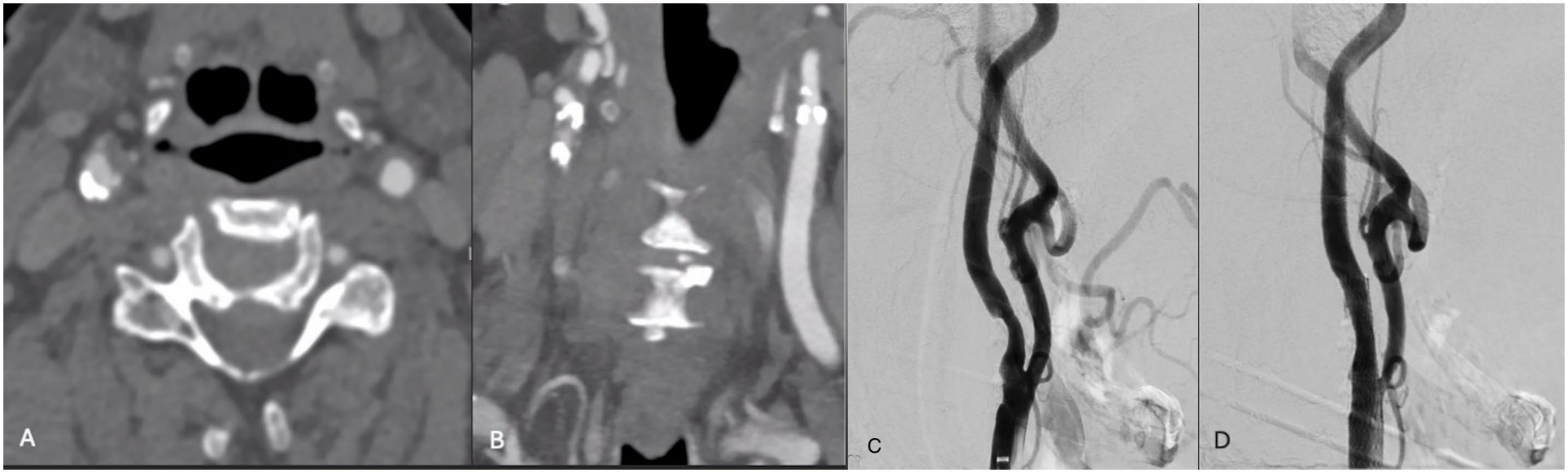
**(A,B)** Axial and coronal CTA demonstrating right ICA circumferential calcified stenosis. Catheter angiography demonstrating **(C)** R ICA stenosis with **(D)** significant improvement in caliber and flow within right ICA following lithotripsy, stent and angioplasty.

A patient in their 80s was hospitalized with an NSTEMI when they developed left-sided facial droop, left-sided paresis, and dysarthria. CTA revealed 50–60% stenosis of the right ICA. MRI revealed punctate foci of infarct in the right frontal and parietal lobes. The patient was discussed with our interdisciplinary neurovascular board, with consensus to pursue CAS with concurrent IVL. The patient was subsequently treated with IVL via 2 episodes of sonic wave emission (Figure 5). Post-angiogram ultrasound showed a PSV of 100 cm/s in the right ICA and a PSV of 193 cm/s in the right ECA with an ICA/CCA ratio of 1.4 on the right. The patient tolerated the procedure well and was discharged in stable condition after 7 days. At follow-up, 2 weeks later, they reported to be doing well and were found to have no neurological deficits.

## Discussion

We present our series of patients with severely calcified symptomatic carotid stenosis treated with IVL and concurrent stenting. All patients showed significant improvement in stenosis following the procedure, with no procedural complications and no debris captured in the distal embolic filter. These findings suggest that Shockwave IVL is a safe concomitant technology for the neurointerventionalists’ armamentarium when treating complex, heavily calcified carotid stenosis in patients not deemed amenable to CEA. While more experience with carotid lithotripsy has been published in the vascular surgery literature (8–13), as it stands, this series also adds to the very limited number of published cases reporting on the use of IVL in the setting of carotid stenosis in the neurointerventional literature. Currently only utilized off-label, this technique highlights the potential for expanding the treatment strategy for cervical carotid artery stenosis.

Shockwave IVL was initially developed to treat renal and ureteral caliculi and has since been expanded to address cardiovascular disease. IVL utilizes a single-use catheter with a 0.014-inch guidewire and a balloon with a length of 12 mm. The central and lateral aspects of the balloon contain emitters designed to release pulsatile pressure waves via fluid vaporization within the balloon (14). Once the catheter has been appropriately placed and the balloon expanded up to 4 atmospheric pressures, these waves are selectively delivered to the calcified lesion. Each emission cycle releases a series of 30 pulsations targeting the calcifications. These shockwaves create serial microfractures in the calcium deposits within the intima and media layers of the vessel wall while preserving the surrounding soft tissue. An increase in impulses has been shown to increase vessel compliance (14). The balloon is then used to dilate the lumen and increase the diameter of the vessel post-intervention.

While urological lithotripsy has been successfully used for several decades, the application of shockwave technology in the cardiovascular system is relatively new and gained FDA approval for calcified peripheral arteries in 2017 and coronary calcifications in 2021. Since then, IVL has been shown to be successful in the treatment of left main artery disease, complete occlusive disease, nodular calcifications, and eccentric calcifications of the coronary arteries in a total of four prospective, multicenter studies (15). Additionally, its use has been validated in the treatment of peripheral artery disease, with success in iliac, femoropopliteal, and infrapopliteal arterial lesions demonstrated in several prospective, multicenter, randomized, and non-randomized trials (8). These data suggest that IVL may be a safer option for managing complicated calcified stenosis, which has led to its exploratory application in carotid calcific disease. The first application of shockwave IVL to a carotid lesion was reported in 2018 for a patient in their 50s with bilateral critical stenosis of the ICA (9). In 2020, Vadalà et al. reported two patients who were not appropriate candidates for vascular surgery due to severe calcifications who were subsequently treated with carotid IVL with good post-procedural stent expansion and 3 patients with similar findings and outcomes were reported in 2024 (10, 16). Additionally, a multicenter case series in 2021 reported on 21 patients treated with IVL followed by CAS across 8 academic centers. All patients had less than 30% stenosis following IVL, and there were no procedural complications; however, long-term follow-up is limited (11). Additionally, vascular surgery has contributed to the literature in reporting the use of adjunctive lithotripsy during transcarotid artery revascularization in 58 patients from 2018 to 2022, with restenosis in 3 patients at follow-up, without thrombosis or fracture (12). Meanwhile, a 2023 paper presents 2 patients, one with restenosis after two carotid endarterectomies (CEAs) and one with previously untreated lesions, who underwent off-label use of IVL for carotid stenosis, both with patent stenting at follow-up (5). This was the first case report of off-label carotid IVL in the neurointerventional literature. The limited data available to date support the use of off-label IVL for severe carotid calcifications in patients who were previously considered candidates for surgery; however, additional data are necessary to broaden IVL indications.

Though still off-label for use in carotid stenosis, IVL addresses an unmet need for treatment strategies in patients with severely calcific carotid stenosis who are not promising candidates for CEA or medical management alone. There are many factors that interfere with a patient’s candidacy for CAS or CEA, including prohibited neck anatomy and comorbid disease. This includes, but is not limited to, high-risk carotid bifurcations, prior radiation or surgical procedures posing challenges with ipsilateral redo surgery, and elderly patients with comorbidities, who face unacceptable risk due to general anesthesia or awake CEA. Additionally, patients may have circumferential calcification, creating challenges for appropriate stent expansion and increasing their risk for complications such as dissection, recoil, and poor angioplasty of the carotid artery. Furthermore, CAS carries a significant risk of restenosis (≥70% stenosis or occlusion) with a 10-year cumulative incidence rate ranging from 5 to 12.2% (13).

Alternative plaque modification techniques, including balloon angioplasty and atherectomy, have been used to treat heavily calcified lesions; however, the risk of vessel injury and distal embolization remains daunting (17). Meanwhile, IVL mitigates these risks by leaving the soft tissue intact and selectively fracturing the medial and intimal plaque. Accordingly, IVL is a promising treatment approach that may be well-suited to addressing this barrier in the field of neurosurgery.

Nevertheless, it is important to consider associated challenges and how they compare to standard treatment options such as medical management, CAS, and CEA. Complications for CEA include cranial nerve injury in 4–16% of cases, and high-risk patient groups have had notably higher prevalence of major stroke, myocardial infarction, or death post treatment (18, 19). CAS has been associated with increased risk of periprocedural stroke, most often secondary to thromboembolism and disruption of hemodynamics (19). Given the mechanism of IVL, the risk of periprocedural stroke secondary to distal emboli is theoretically the same. As discussed, lithotripsy expels pressure waves, fracturing the calcium deposits multidimensionally in the intimal and medial layers of the vessel while leaving the surrounding tissues undisturbed without peripheral circulation of calcium. Therefore, the likelihood of distal embolization is particularly low. The risk of periprocedural vessel perforation is also low, as the mechanical stress on the tissue is minimized due to the mild balloon pressure maintained throughout the procedure (20). Of note, IVL requires longer balloon inflation than conventional angioplasty, increasing the patient’s risk of baroreceptor disturbance, which can lead to bradycardia or asystole. Therefore, having atropine or glycopyrrolate on board may be an appropriate measure as demonstrated in Mehta and Wooster’s report, and this approach was utilized in three of our patients at the anesthesiologist’s discretion (10).

Given the small size of this series, limitations include the possibility of selection bias. The data reported in this study are preliminary, and long-term follow-up remains necessary. Generalizability is limited as this is a single-center study with a small number of patients, and all patients underwent treatment by the same physician. The data must be further elucidated to assess the feasibility of femoral versus radial artery access and the ideal ultrasonic pulse paradigm to achieve maximal radiographic benefit. The occurrence of radiographic embolic events was not documented in this study, as there was no standard MRI with diffusion-weighted imaging (DWI) across all patients following the IVL procedure.

## Conclusion

In this preliminary proof-of-concept case series, IVL has shown promising results in patients with significant carotid artery calcification who are not amenable to standard surgery. Further observation of the patients in this series and additional experience with IVL at our center will inform the data regarding long-term outcomes and complications. Large, prospective studies are warranted to determine the safety and efficacy in a larger population and increase generalizability with long-term follow-up. Additionally, future investigations may explore objective measures of neurological improvement to better quantify the benefit seen in this population.

## Data Availability

The original contributions presented in the study are included in the article/supplementary material, further inquiries can be directed to the corresponding author.
